# Investigating tryptophan metabolism in colorectal cancer using Single-cell RNA sequencing based on machine learning techniques

**DOI:** 10.1371/journal.pone.0352871

**Published:** 2026-07-06

**Authors:** Chen Zepeng, Wang Xingchen, Xiao Changfang, Cao Yongqing

**Affiliations:** 1 Department of Anorectal Surgery, LongHua Hospital Shanghai University of Traditional Chinese Medicine, Shanghai, China; 2 Shanghai University of Traditional Chinese Medicine, Shanghai, China; University of Pennsylvania Perelman School of Medicine, UNITED STATES OF AMERICA

## Abstract

**Background:**

Colorectal cancer (CRC) is characterized by genetic variation, epigenetic alterations, microenvironmental imbalance, and metabolic reprogramming. Currently, abnormalities amino acid metabolism has been shown to play an important role in the occurrence and progression of CRC.

**Methods:**

AUCell, UCell, singscore, ssGSEA and AddModuleScore algorithms were used to determine the pattern of tryptophan metabolism in CRC at the cellular level. Differential expression and correlation analyses were performed to identify core candidate genes associated with upregulation of metabolic activity. Four machine learning algorithms——random forest, Boruta, LASSO, and gradient boosting machine—were further integrated for feature selection. Finally, to enhance robustness and reduce algorithm‑specific bias, the results of these algorithms were combined to identify the key feature genes related to tryptophan metabolism in CRC.

**Results:**

The findings demonstrated significant differences in tryptophan metabolic activity among different cell types in CRC, with macrophages and Paneth cells exhibiting higher activity. Among the tryptophan metabolism-related genes, *CYP1A1* and aryl hydrocarbon receptor (*AHR*) were significantly upregulated in CRC, suggesting their involvement in regulating of immune response and inflammatory responses.

**Conclusions:**

This study reveals, for the first time, the cellular pattern of tryptophan metabolism in CRC, with macrophages and Paneth cells playing a major role in tumor development. *CYP1A1* and *AHR* were identified as consensus feature‑selected genes involved in tryptophan metabolism in CRC, highlighting their potential as biomarkers and therapeutic targets.

## Introduction

Colorectal cancer (CRC) is one of the most common malignant tumors worldwide, and its disease burden has changed significantly in recent decades. In China, its incidence ranks second among all malignant tumors [1]. Its occurrence and progression represent a complex process involving genetic variation, epigenetic alterations, microenvironmental imbalance, and metabolic reprogramming [2,3]. In recent years, abnormalities in tumor metabolism—particularly in amino acid metabolism—have been shown to be deeply involved in tumor immune escape, proliferation, and metastasis [4]. Among these processes, the metabolic regulatory network of tryptophan, an essential amino acid, plays a particularly critical and multifaceted role in the progression of colon cancer and has become an important bridge linking the intrinsic characteristics of tumor cells with the dynamic changes in the immune microenvironment [5,6]. However, the specific cellular mechanisms and genetic factors that regulate tryptophan metabolism remain largely unexplored.

Tryptophan metabolism occurs through three major pathways: the kynurenine pathway, the serotonin pathway, and the microbial indole–generation pathway [7]. In colon cancer, aberrant activation of the kynurenine pathway has been studied most extensively. The rate-limiting enzymes indoleamine 2, 3-dioxygenase 1 and tryptophan 2, 3-dioxygenase are highly expressed in tumor cells, myeloid cells (such as dendritic cells and macrophages), and certain stromal cells within the tumor microenvironment [8]. Activation of these enzymes results in substantial local depletion of tryptophan and the concomitant accumulation of metabolites such as kynurenine. This process exerts a dual impact on the tumor immune microenvironment. In contrast, tryptophan depletion activates amino acid–sensing pathways (such as the GCN2 kinase pathway), thereby inhibiting the proliferation and function of effector T cells and inducing apoptosis. Conversely, accumulated kynurenine metabolites directly promote the differentiation and function of regulatory T cells (Tregs) through activation of the aryl hydrocarbon receptor (AhR) and related pathways, while suppressing antitumor immune responses mediated by natural killer cells. Collectively, these effects create a highly immunosuppressive microenvironment that facilitates tumor immune escape [9,10].

In addition to immune regulation, tryptophan metabolites can directly influence the fate of tumor cells. Sustained activation of AhR has been shown to promote proliferation, maintenance of stemness, and resistance to chemotherapy in colon cancer cells [11]. Moreover, indole and its derivatives (such as IPA and IAA), which are produced by the intestinal microbiota during tryptophan metabolism, exhibit more complex effects. Some microbiota-derived metabolites exert anti-inflammatory effects and protect the intestinal mucosal barrier, thereby potentially inhibiting tumorigenesis, whereas others may promote inflammation or genotoxicity under specific conditions and indirectly influence carcinogenesis [12,13]. These findings suggest that host–microbe interactions in tryptophan metabolism represent an important factor in determining local homeostasis or malignant transformation in the colon.

To our knowledge, this study is the first to reveal the single-cell heterogeneity of tryptophan metabolism in CRC and demonstrates that metabolic activity varies significantly among different cell types. By integrating functional enrichment analysis with machine learning approaches, we further identified key factors associated with tryptophan metabolism that may drive the pathological progression of CRC and therefore serve as potential therapeutic targets. These findings provide an important foundation for future CRC research and the development of targeted treatment strategies.

## Methods

### Single-cell RNA-seq data sources and preprocessing

This study obtained CRC single-cell RNA sequencing (scRNA-seq) data from the Gene Expression Omnibus database (https://www.ncbi.nlm.nih.gov/geo/). Specifically, three datasets were included: GSE289314, GSE288119, and GSE161277, comprising a total of 14 CRC patient samples and 13 healthy control samples. All datasets were derived from publicly available CRC-related scRNA-seq studies and contained single-cell transcriptomic data from colonic mucosal tissues. Tryptophan metabolism–related genes (TrMGs) were identified based on the Kyoto Encyclopedia of Genes and Genomes and Gene Ontology (GO) databases, as well as relevant published literature. Ultimately, 27 genes were included for subsequent analyses [8,14–17]. During scRNA-seq data processing, we applied the following quality control criteria: (1) cells with a mitochondrial gene proportion exceeding 20% were excluded to remove dead or damaged cells; (2) cells with fewer than 200 detected genes were excluded to eliminate empty droplets or low-quality cells; and (3) only cells with 200–6000 expressed genes were retained to exclude potential doublets or cells with abnormally high expression levels. After the above quality control procedures, a total of 58,089 qualified cells were retained for downstream analyses [18]. Doublets were identified by the 10x Genomics Cell Ranger pipeline during the cellranger count step. The algorithm uses the expected multiplet rate (provided by 10x based on the number of loaded cells) and a simulated doublet model to classify each cell as singlet or doublet. Only cells labeled as singlet were retained for downstream analysis. Subsequently, expression values were normalized using the log-normalization method, and the expression matrix was scaled using the “ScaleData” function with linear regression to mitigate the effects of mitochondrial gene proportion and cell cycle heterogeneity. Additionally, the “FindVariableFeatures” function was applied to identify the top 3,000 highly variable genes. Principal component analysis was performed to reduce data dimensionality. Cell type annotation was performed using the R package “SingleR,” with the Human Primary Cell Atlas and Blueprint/ENCODE reference datasets. This step integrated the results of uniform manifold approximation and projection and dataset integration [19]. To correct batch effects among samples, the “Harmony” algorithm was applied, which uses iterative soft k-means clustering and linear correction to remove batch effects while preserving biological variation [20]. The parameters were set as follows: theta = 2 (clustering diversity penalty parameter) and nIter = 20 (maximum number of iterations). Cell clustering was performed using the “FindClusters” function, with the resolution parameter set to 0.8. This resolution was chosen because it is a widely adopted parameter in the literature [21,22]. The effectiveness of batch correction was quantitatively evaluated using the Local Inverse Simpson‘s Index (LISI), which estimates the effective number of batch classes in local neighborhoods of cells after correction. For each cell, we computed the integration LISI (iLISI) as a measure of batch mixing. The cell‑type LISI (cLISI) was also calculated to assess whether cell‑type identity remained intact after correction. Both metrics were implemented using the lisi R package. Cell clusters were identified based on highly expressed genes, distinct expression patterns, and established classical cell markers.

### Bulk RNA-seq data sources and processing

To validate the expression patterns of differentially expressed genes identified by scRNA-seq in a larger cohort, we further analyzed two independent bulk RNA-seq datasets. Two datasets were selected to ensure comparable proportions of tumor and normal samples. Importantly, the bulk RNA-seq data used in this study were derived from entirely independent patient cohorts and were not matched to the scRNA-seq samples. After screening, GSE21815 was designated as the training set, and GSE103512 was used as the validation set (http://www.ncbi.nlm.nih.gov/geo/). The two datasets were selected based on sample size, completeness of clinical information, and consistency of sequencing platforms to ensure their suitability as training and validation sets. All bulk RNA-seq data underwent normalization (log2 transformation) and batch effect correction before being used for subsequent differential expression analysis and machine learning modeling. This dual-dataset design of “bulk discovery–single-cell validation” was employed to leverage the advantages of both the large sample size of bulk RNA-seq, which provides high statistical power for gene screening, and the high resolution of single-cell RNA-seq, which enables precise identification of the cellular origins of gene expression [23,24].

### Gene set scoring methods in single-cell data

To evaluate gene set activity in the scRNA-seq data, we applied five scoring algorithms: AUCell [25], UCell [26], singscore [27], ssGSEA [28], and AddModuleScore [29]. Among these, AUCell and UCell were selected as primary methods because of their ability to accurately quantify gene set activity at the single-cell level, which is essential for identifying activation patterns in CRC cells. AUCell evaluates gene set activity in each cell by calculating the area under the cumulative distribution curve (AUC) based on gene expression rankings. UCell computes a standardized ranking score according to single-cell gene expression rankings. Singscore ranks genes within a predefined gene set for each cell and derives a score based on the difference between the mean ranks of positive and negative genes. ssGSEA calculates a relative enrichment score by comparing the expression values of genes within the target gene set to those of the remaining genes. AddModuleScore computes the average expression level of genes in the specified gene set and normalizes the result to obtain a score for each cell.

### Differential expression analysis and functional annotation

The “FindMarkers” function was used to identify differentially expressed genes (DEGs) between the high and low TrMG expression groups. The screening thresholds were set at |log2 fold change| > 0.25 and an adjusted p value < 0.05. This relatively lenient threshold was selected to capture subtle yet potentially biologically meaningful changes in single-cell data, where expression differences may be attenuated because of technical noise and cellular heterogeneity. In addition, to identify genes most closely associated with TrMG expression, correlation analysis was performed, and the top 100 genes with the highest correlation coefficients were selected for subsequent analyses. These DEGs and genes significantly associated with TrMG expression were further used for mechanistic exploration. GO enrichment analysis was conducted on these genes using the “clusterProfiler” package in R to elucidate their potential biological functions and pathways.

### Machine learning–based screening of candidate TrMGs

To identify robust tryptophan metabolism–related hub genes in CRC, we performed an exploratory feature selection analysis using four complementary machine learning algorithms: the Boruta algorithm [30], LASSO [31], gradient boosting machine (GBM) [32] and random forest [33]. This analysis was conducted for variable screening. To enhance the stability of feature selection against data perturbations, five-fold cross-validation was implemented in the training set. Specifically, the training set was equally divided into five folds; in each iteration, four folds were used for feature selection and the remaining fold was used for validation, and this process was repeated five times. The response variable of all algorithms was the binary disease status (CRC vs. normal). In the Boruta algorithm, the parameters were set as maxRuns = 100 (maximum number of iterations) and a significance threshold of p < 0.01 for confirming or rejecting features. Only features confirmed as “important” in all cross‑validation folds were considered candidate genes. LASSO regression was performed using the “glmnet” package, applying regularization to shrink regression coefficients and thereby select candidate genes while eliminating redundant genes. The GBM method iteratively constructed decision trees, each correcting the errors of the previous one. Random forest constructed multiple decision trees and aggregated their results to evaluate the importance of each gene, after which the top 20 candidate genes were selected. To increase robustness and reduce algorithm‑specific bias, only the genes that were consistently selected by all four algorithms across the cross-validation folds were retained as the final set of core TrMGs. This intersection strategy has been widely used to improve the stability and reproducibility of feature selection in high‑dimensional transcriptomic studies [34,35]. The intersection results were visualized using a Venn diagram. The objective of this analysis is strictly exploratory – to identify a compact set of high‑confidence candidate genes for downstream biological interpretation, not to build or validate any predictive model.

### Statistical Analysis

All statistical analyses and visualizations were performed using R version 4.1.3. Differences in continuous variables between groups were analyzed using the Wilcoxon rank-sum test or Student’s t test, whereas categorical variables were compared using the chi-square test or Fisher’s exact test. All statistical analyses were conducted using two-sided tests, and a p value < 0.05 was considered statistically significant.

## Results

### scRNA-seq profiling of CRC

**Fig 1:**
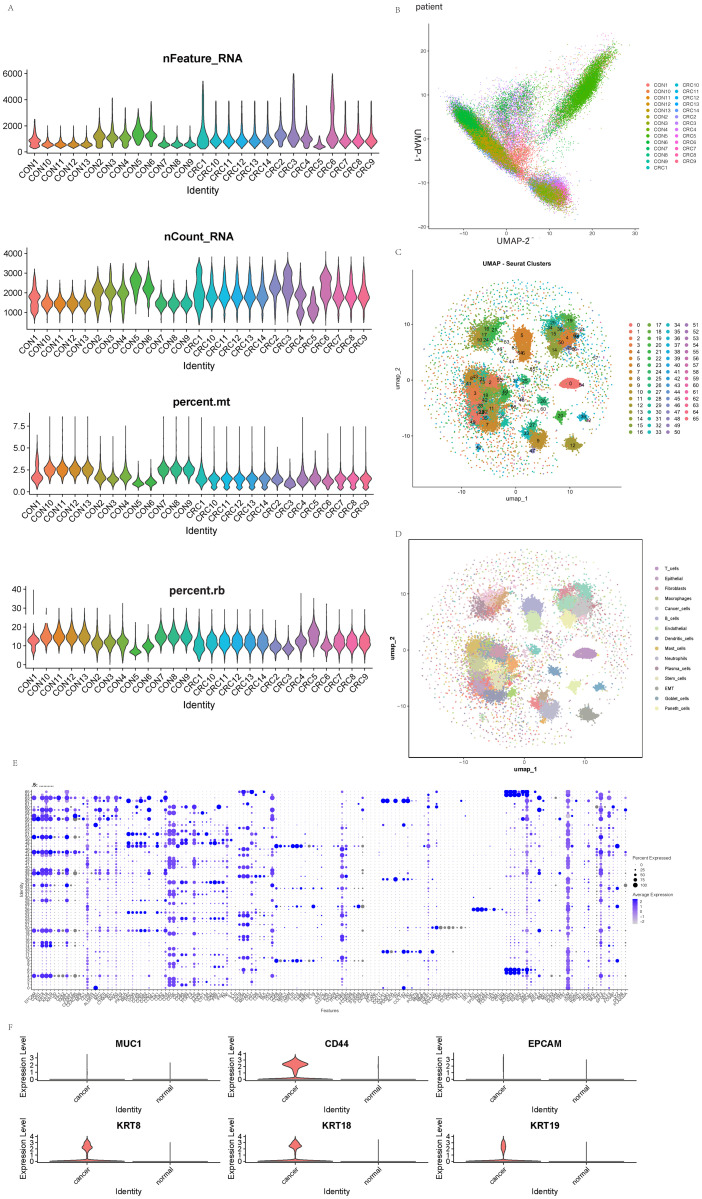
Explanation of cellular subpopulations. (A) Quality control for inclusive data. (B) Excluding batch effects between samples. (C) Seurat clusters of eligible cells in umap plot. (D) Cellular annotations unveil distinct cell phenotypes. (E) Bubble plot of relative expression of marker genes for each cell type. (F) UMAP plot reveals marker gene expression levels across diverse cell types.

A total of 27 samples were included in the analysis, comprising 14 CRC samples and 13 control (CON) samples. Before in-depth analysis, all samples underwent stringent quality control (Fig 1A) and batch effect correction (Fig 1B). The results indicated that the overall data distribution was stable and that batch effects were minimal. Following analysis using the standard Seurat workflow, all cells were classified into 65 clusters, as shown in the detailed clustering results (Fig 1C), UMAP visualizations before correction are shown in Supplement Figure1. The iLISI score increased from 1.2 to 6.617, higher iLISI values indicate more complete mixing across batches; the cLISI score decreased from 2.1 to 1.12, values close to 1 indicate minimal disruption of biological cell types. indicating improved mixing across batches. Subsequently, the expression patterns of characteristic marker genes for each cell subpopulation were visualized (Fig 1D), and cell types were annotated based on established specific marker genes (Fig 1E). In addition, several representative genes were selected for further validation of cell type classification (Fig 1F). The immune response plays an important role in the pathogenesis of CRC.

### Evaluation of tryptophan metabolism in single-cell data

**Fig 2:**
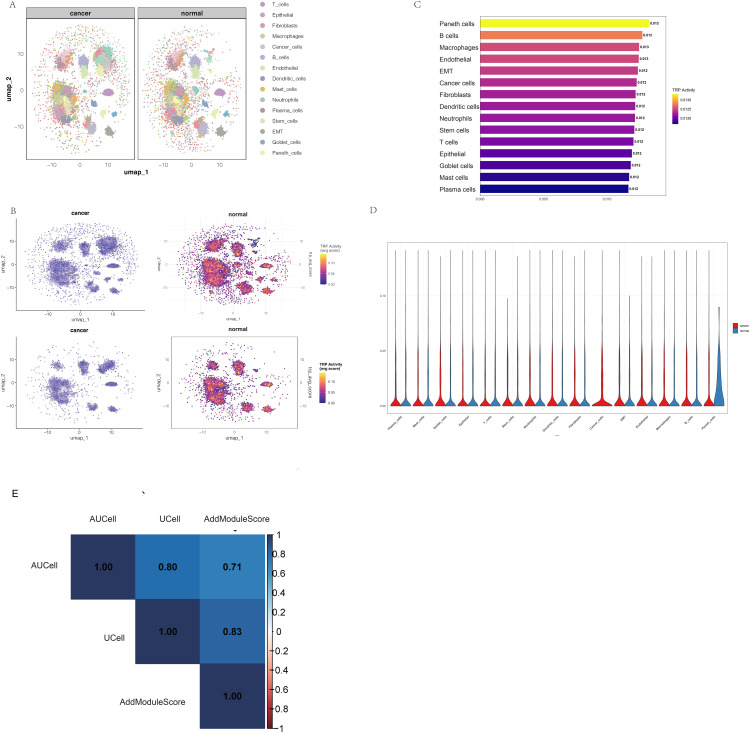
Heterogeneity among the expression of TrMGs. (A) Celltype of tissues; (B) TrMG expression of tissues; (C) showed expression scores of TrMGs for each cell type using AUCell, UCell, singscore, ssGSEA, and Add algorithms; (D) Violin plot showed the difference in TrMGs score of the CON and CRC groups; (E) Correlation analysis among AUCell, UCell, and AddModuleScore.

The tryptophan metabolic pathway plays an important role in the progression of CRC, and its activity is dysregulated in patients with CRC. To evaluate tryptophan metabolic activity at the single-cell level, we comprehensively applied five algorithms: AUCell, UCell, and AddModuleScore. The activity of TrMGs across different cell types was assessed by calculating their average pathway scores (Fig 2A-2B). The analysis revealed that TrMG activity was highest in macrophages and Paneth cells but relatively lower in goblet cells, T cells, and epithelial cells (Fig 2C). Further comparison between the CRC and CON groups demonstrated that TrMGs were upregulated in tumor cells and Paneth cells, whereas they were downregulated in stem cells and fibroblasts in the CRC group (Fig 2D). To evaluate the robustness of pathway activity defined by the TrMG gene set across different algorithms, pathway activity scores were calculated for all 58,089 cells using using the AUCell, UCell, and AddModuleScore algorithms, followed by pairwise correlation analyses. The results demonstrated significant positive correlations among all algorithms (Spearman correlation coefficients of 0.8). Specifically, the correlation coefficient between AUCell and UCell was 0.80, between AUCell and AddModuleScore was 0.83, and between UCell and AddModuleScore was 0.71 (all P < 0.001) (Fig 2E) (S1 Table). These findings indicate that despite the different mathematical principles underlying each algorithm, their assessments of TrMG pathway activity are highly consistent, confirming that the biological signals captured by the gene set defined in this study are robust and reproducible. This result provides a reliable methodological foundation for subsequent cell-type-specific analyses based on these scores.

### Overlapping gene analysis based on multi-source data

**Fig 3:**
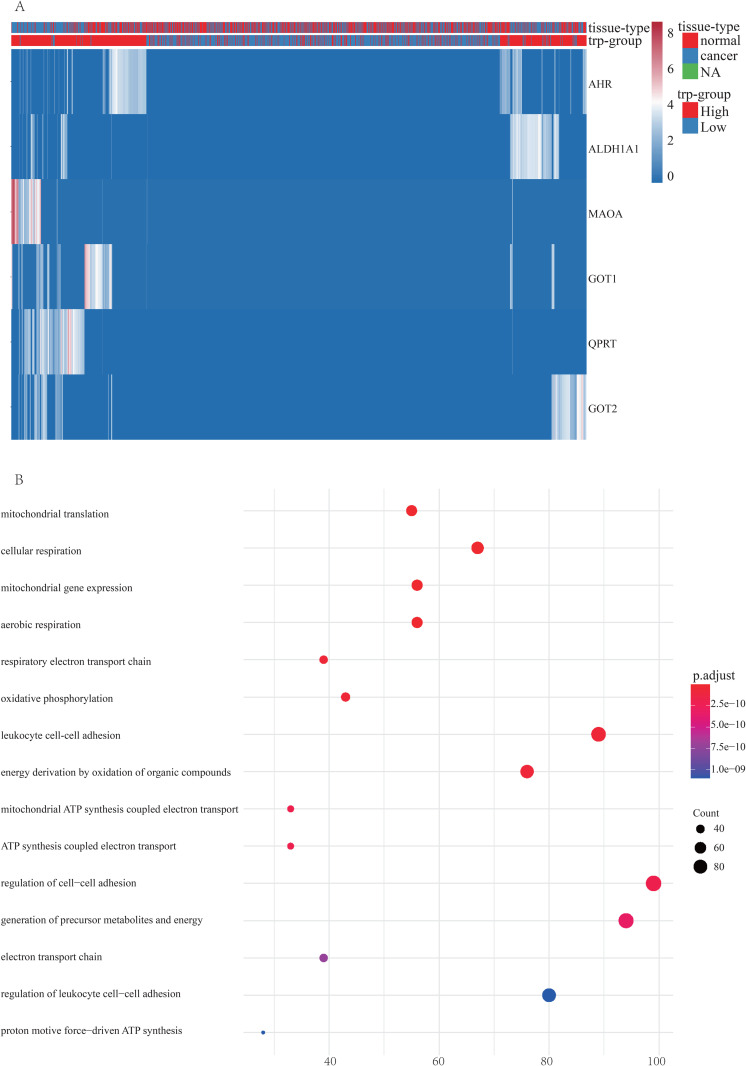
Cross analysis of the key genes based on bulk data. (A) six genes were up regulated in the CRC group; (B) The heatmap of overlapping genes expression.

To further evaluate the reliability of the 27 TrMGs, we conducted integrative analyses using multi-source transcriptomic data. By cross-comparing the key genes identified from the scRNA-seq data with those identified in the bulk RNA-seq datasets (S1 Table), six genes—*AHR, ALDH1A1, MAOA, GOT1, QPRT,* and *GOT2*—were consistently upregulated in the CRC group (Fig 3A). These findings were further confirmed by heatmap visualization. GO enrichment analysis demonstrated that the 27 genes were closely associated with specific biological processes (S2 Table), particularly immune and inflammatory responses (Fig 3B).

### Identification of the optimal genes by machine learning

**Fig 4:**
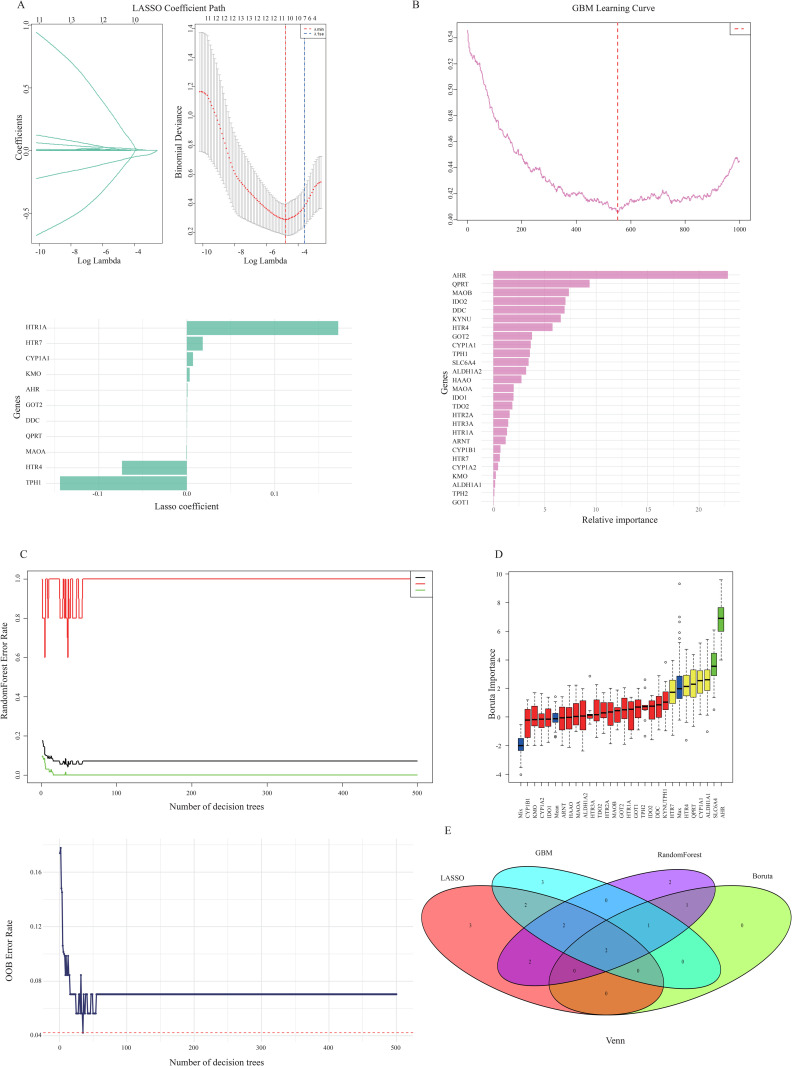
Identification of the marker genes by using machine learning. (A-D) The LASSO algorithm (A), GBM algorithm (B), random forest (C), Boruta algorithm (D) determined the candidate optimal feature genes. (E) Venn diagram displayed the two optimal key genes overlapped by the four above-mentioned.

Four machine learning algorithms—LASSO (Fig 4A), GBM (Fig 4B), random forest (Fig 4C), and Boruta (Fig 4D) —were applied to identify the most relevant candidate feature genes in the training set. Cross-analysis of genes consistently selected by these algorithms identified two signature genes: *CYP1A1* and *AHR* (Fig 4E).

## Conclusion

Dysregulation of tryptophan metabolism has been recognized as an important factor in the development of CRC. Previous studies have shown that tryptophan metabolism, particularly the kynurenine–*AHR* axis, has emerged as a promising therapeutic target in CRC [36–39]. However, the specific regulatory mechanisms and key associated genes remain unclear. Therefore, this study integrated scRNA-seq technology with machine learning algorithms to investigate the complex interactions underlying tryptophan metabolism in CRC. This integrative approach enabled a more precise characterization of the metabolic profiles of distinct cell subsets in CRC.

We identified two key genes, *CYP1A1* and *AHR*, that were significantly upregulated in CRC and were closely associated with dysregulation of the tryptophan metabolic pathway. By integrating single‑cell RNA‑seq with machine learning algorithms, we increased the robustness of gene selection and reduced algorithm‑specific bias. The identified genes are not only statistically significant but also biologically relevant to CRC pathogenesis. The identification of these genes provides new insights into the molecular mechanisms underlying CRC and suggests potential targets for therapeutic intervention.

The *CYP1A1* gene encodes a member of the cytochrome P450 family, which is primarily involved in drug metabolism and carcinogen detoxification. Its mechanism of action involves enzymatic catalytic activity, particularly in the metabolism of polycyclic aromatic hydrocarbons (PAHs), such as benzo[a]pyrene, through oxidative reactions that convert these compounds into more water-soluble metabolites for excretion [40]. *AHR* belongs to the basic helix–loop–helix Per-ARNT-Sim superfamily and functions as a ligand-dependent transcription factor that mediates the toxic effects of halogenated hydrocarbons, including dioxins [41,42]. It regulates gene transcription through both classical and nonclassical signaling pathways and participates in xenobiotic metabolism and immune responses. AhR exerts multiple functions in the immune system. Upregulation of *AHR* expression regulates memory differentiation of CD8^+^ T cells through the ROS–Nrf2 pathway, and sustained activation promotes a metabolic shift toward fatty acid oxidation while maintaining the IL-2–STAT5–TPH1–5-HTP signaling pathway [43]. Abnormal activation of AhR in the tumor microenvironment leads to an imbalance in megakaryocyte–erythroid progenitor differentiation, promoting platelet production while inhibiting erythrocyte differentiation through the Kyn–RUNX1 signaling pathway [44]. PAHs, tryptophan metabolites (such as FICZ and kynurenine), and certain gut microbiota–derived metabolites act as ligands that activate AhR. Upon activation, AhR translocates into the nucleus and induces the expression of a series of target genes, including *CYP1A1* [45,46]. The expression level of *CYP1A1* is frequently used as a biomarker of AhR pathway activation. AhR activation directly regulates the differentiation and function of multiple immune cell types, promoting the generation and suppressive function of Treg cells and thereby attenuating inflammatory and immune responses [47]. In the presence of specific ligands, such as FICZ, AhR can also promote the differentiation of proinflammatory T helper 17 cells and participate in mucosal immunity and autoimmune responses [48]. Moreover, AhR regulates the function of group 3 innate lymphoid cells and influences interleukin-22 production, thereby maintaining intestinal barrier integrity [49].

In CRC, the role of the *CYP1A1*–AhR pathway may either inhibit tumorigenesis or promote tumor progression, depending on the tumor microenvironment, the nature of the activating ligand, and the disease stage. *CYP1A1* metabolizes and activates various dietary and environmental procarcinogens (such as polycyclic aromatic hydrocarbons and heterocyclic amines), converting them into more water-soluble forms that are readily excreted, thereby reducing the risk of DNA damage and carcinogenesis [50]. AhR activation enhances intestinal epithelial barrier function and suppresses chronic inflammation—an important driver of CRC—thus creating a microenvironment that is unfavorable for tumor initiation [51]. Some studies have reported that high *CYP1A1* expression is associated with poor prognosis in patients with CRC, possibly reflecting persistent AhR activation and immunosuppression. However, other studies have demonstrated an association with improved prognosis, which may reflect its detoxification function [52]. These discrepancies may be related to differences in patient populations, dietary patterns, and environmental exposures. The AhR–*CYP1A1* axis represents a potential therapeutic target, as inhibition of AhR may reverse immunosuppression within the tumor microenvironment and enhance the efficacy of immune checkpoint inhibitors, such as anti–PD-1 antibodies [53,54].

Another notable finding was that TrMG activity exhibited substantial heterogeneity across different cell types, with macrophages and Paneth cells showing significantly elevated tryptophan metabolic activity. This observation is consistent with metabolic–immune mechanisms reported in multiple recent studies, suggesting that these two cell types may act as synergistic drivers within a tryptophan metabolism–remodeled tumor microenvironment. A recent multi-omics study confirmed that tryptophan metabolism is significantly enhanced in CRC tissues, leading to the accumulation of metabolites such as kynurenine, and that this metabolic feature is positively correlated with M2 macrophage infiltration [17]. The underlying mechanism may involve activation of the AhR pathway. Previous studies have shown that tryptophan metabolites promote polarization of tumor-associated macrophages toward the M2 phenotype via AhR signaling, thereby suppressing antitumor immunity [15]. Paneth cells, as key regulators of the intestinal stem cell niche, are functionally influenced by AhR signaling [55]. The spatial proximity of Paneth cells to stem cells, together with their secretion of antimicrobial peptides, suggests that alterations in their metabolic state may influence tumorigenesis through two mechanisms: first, by directly acting on adjacent cancer stem cells through metabolites (such as kynurenine) to promote stemness maintenance; and second, by indirectly reshaping the composition of the gut microbiota through changes in antimicrobial peptide secretion, thereby modulating the tumor microenvironment via the microbiota–metabolism axis [56]. Recent studies have further demonstrated that gut microbiota–derived tryptophan metabolites significantly influence the response of CRC to chemotherapy, and this process is dependent on macrophage-mediated mechanisms. These findings suggest that Paneth cells may serve as a critical node linking host metabolism, the gut microbiota, and immune cells.

This study also found that macrophage signature genes, such as *LSP1* and *ARHGDIB*, were upregulated in CRC, consistent with previous reports of increased tumor-associated macrophage infiltration [57]. Notably, some of these genes (e.g., *RAC2*) have been reported to participate in regulation of the AhR signaling pathway. Studies have shown that *RAC2*, a hematopoietic cell–specific small GTPase, plays a key role in macrophage phagocytosis, cytoskeletal remodeling, and immunomodulation. A recent single-cell study further revealed that *RAC2*-mediated macrophage efferocytosis can drive M2 polarization and promote formation of an immunosuppressive microenvironment [58]. Furthermore, *RAC2* is involved in regulating COX-2 expression and the NF-κB signaling pathway in macrophages, closely linking it to inflammatory responses [59]. These findings suggest that tryptophan metabolism may regulate antitumor immune responses by influencing *RAC2*-mediated macrophage cytoskeletal remodeling and phagocytic function. Targeting this metabolic–immune regulatory axis may represent a novel therapeutic strategy for CRC.

Our study also has some limitations. First, the expression levels of tryptophan metabolism–related genes may be relatively low and therefore easily missed in scRNA-seq analysis, which may hinder comprehensive reconstruction of metabolic pathways. Moreover, scRNA-seq measures gene expression but cannot directly reflect metabolite concentrations or metabolic flux; thus, tryptophan metabolic activity requires further validation through metabolomic analyses. Second, annotations of tryptophan metabolism in existing databases may lack cell type specificity, making it difficult to accurately assign metabolic features to specific tumor cell subsets. In addition, the sparsity of the gene expression matrix may result in loss of information on key metabolic enzyme genes, thereby affecting the model’s ability to predict metabolic status. Therefore, future studies should integrate spatial transcriptomics, single-cell metabolomics, and other complementary technologies to validate metabolic regulatory mechanisms from multiple dimensions. Expanding patient cohorts and incorporating longitudinal sample analyses will also be important to evaluate dynamic changes in tryptophan metabolism in response to CRC treatments, such as immunotherapy.

## Supporting information

S1 Table Differential expression. (CSV)

S2 Table GO enrichment. (CSV)

S1 File Supplment R. (DOCX)
