# Supplementary material for: Investigating tryptophan metabolism in colorectal cancer using Single-cell RNA sequencing based on machine learning techniques
Source: PLoS One. 2026 Jul 6;21(7):e0352871. doi: 10.1371/journal.pone.0352871 (PMC13336214; doi:10.1371/journal.pone.0352871)
Supplement: S1 File — (DOCX) [file pone.0352871.s003.docx]

setwd("...")

getwd()

install.packages("Seurat")

install.packages("promises")

remove.packages("promises")

library(Seurat)

cancer1_dir <- ".../dir/cancer1"

cancer2_dir <- ".../dir/cancer2"

cancer3_dir <- ".../dir/cancer3"

cancer4_dir <- ".../dir/cancer4"

cancer5_dir <- ".../dir/cancer5"

cancer6_dir <- ".../dir/cancer6"

cancer7_dir <- ".../dir/cancer7"

cancer8_dir <- ".../dir/cancer8"

cancer9_dir <- ".../dir/cancer9"

cancer10_dir <- ".../dir/cancer10"

cancer11_dir <- ".../dir/cancer11"

cancer12_dir <- ".../dir/cancer12"

cancer13_dir <- ".../dir/cancer13"

cancer14_dir <- ".../dir/cancer14"

normal1_dir <- ".../dir/normal1"

normal2_dir <- ".../dir/normal2"

normal3_dir <- ".../dir/normal3"

normal4_dir <- ".../dir/normal4"

normal5_dir <- ".../dir/normal5"

normal6_dir <- ".../dir/normal6"

normal7_dir <- ".../dir/normal7"

normal8_dir <- ".../dir/normal8"

normal9_dir <- ".../dir/normal9"

normal10_dir <- ".../dir/normal10"

normal11_dir <- ".../dir/normal11"

normal12_dir <- ".../dir/normal12"

normal13_dir <- ".../dir/normal13"

list.files(cancer1_dir)

list.files(normal1_dir)

cancer1_data <- Read10X(data.dir = cancer1_dir, gene.column = 1)

cancer2_data <- Read10X(data.dir = cancer2_dir, gene.column = 1)

cancer3_data <- Read10X(data.dir = cancer3_dir, gene.column = 1)

cancer4_data <- Read10X(data.dir = cancer4_dir, gene.column = 1)

cancer5_data <- Read10X(data.dir = cancer5_dir, gene.column = 1)

cancer6_data <- Read10X(data.dir = cancer6_dir, gene.column = 1)

cancer7_data <- Read10X(data.dir = cancer7_dir, gene.column = 1)

cancer8_data <- Read10X(data.dir = cancer8_dir, gene.column = 1)

cancer9_data <- Read10X(data.dir = cancer9_dir, gene.column = 1)

cancer10_data <- Read10X(data.dir = cancer10_dir, gene.column = 1)

cancer11_data <- Read10X(data.dir = cancer11_dir, gene.column = 1)

cancer12_data <- Read10X(data.dir = cancer12_dir, gene.column = 1)

cancer13_data <- Read10X(data.dir = cancer13_dir, gene.column = 1)

cancer14_data <- Read10X(data.dir = cancer14_dir, gene.column = 1)

normal1_data <- Read10X(data.dir = normal1_dir, gene.column = 1)

normal2_data <- Read10X(data.dir = normal2_dir, gene.column = 1)

normal3_data <- Read10X(data.dir = normal3_dir, gene.column = 1)

normal4_data <- Read10X(data.dir = normal4_dir, gene.column = 1)

normal5_data <- Read10X(data.dir = normal5_dir, gene.column = 1)

normal6_data <- Read10X(data.dir = normal6_dir, gene.column = 1)

normal7_data <- Read10X(data.dir = normal7_dir, gene.column = 1)

normal8_data <- Read10X(data.dir = normal8_dir, gene.column = 1)

normal9_data <- Read10X(data.dir = normal9_dir, gene.column = 1)

normal10_data <- Read10X(data.dir = normal10_dir, gene.column = 1)

normal11_data <- Read10X(data.dir = normal11_dir, gene.column = 1)

normal12_data <- Read10X(data.dir = normal12_dir, gene.column = 1)

normal13_data <- Read10X(data.dir = normal13_dir, gene.column = 1)

seurat_cancer1 <- CreateSeuratObject(counts = cancer1_data,

project = "CRC1",

min.cells = 3,

min.features = 200)

seurat_cancer2 <- CreateSeuratObject(counts = cancer2_data,

project = "CRC2",

min.cells = 3,

min.features = 200)

seurat_cancer3 <- CreateSeuratObject(counts = cancer3_data,

project = "CRC3",

min.cells = 3,

min.features = 200)

seurat_cancer4 <- CreateSeuratObject(counts = cancer4_data,

project = "CRC4",

min.cells = 3,

min.features = 200)

seurat_cancer5 <- CreateSeuratObject(counts = cancer5_data,

project = "CRC5",

min.cells = 3,

min.features = 200)

seurat_cancer6 <- CreateSeuratObject(counts = cancer6_data,

project = "CRC6",

min.cells = 3,

min.features = 200)

seurat_cancer7 <- CreateSeuratObject(counts = cancer7_data,

project = "CRC7",

min.cells = 3,

min.features = 200)

seurat_cancer8 <- CreateSeuratObject(counts = cancer7_data,

project = "CRC8",

min.cells = 3,

min.features = 200)

seurat_cancer9 <- CreateSeuratObject(counts = cancer7_data,

project = "CRC9",

min.cells = 3,

min.features = 200)

seurat_cancer10 <- CreateSeuratObject(counts = cancer7_data,

project = "CRC10",

min.cells = 3,

min.features = 200)

seurat_cancer11 <- CreateSeuratObject(counts = cancer7_data,

project = "CRC11",

min.cells = 3,

min.features = 200)

seurat_cancer12 <- CreateSeuratObject(counts = cancer7_data,

project = "CRC12",

min.cells = 3,

min.features = 200)

seurat_cancer13 <- CreateSeuratObject(counts = cancer7_data,

project = "CRC13",

min.cells = 3,

min.features = 200)

seurat_cancer14 <- CreateSeuratObject(counts = cancer7_data,

project = "CRC14",

min.cells = 3,

min.features = 200)

seurat_normal1 <- CreateSeuratObject(counts = normal1_data,

project = "CON1",

min.cells = 3,

min.features = 200)

seurat_normal2 <- CreateSeuratObject(counts = normal2_data,

project = "CON2",

min.cells = 3,

min.features = 200)

seurat_normal3 <- CreateSeuratObject(counts = normal3_data,

project = "CON3",

min.cells = 3,

min.features = 200)

seurat_normal4 <- CreateSeuratObject(counts = normal4_data,

project = "CON4",

min.cells = 3,

min.features = 200)

seurat_normal5 <- CreateSeuratObject(counts = normal5_data,

project = "CON5",

min.cells = 3,

min.features = 200)

seurat_normal6 <- CreateSeuratObject(counts = normal6_data,

project = "CON6",

min.cells = 3,

min.features = 200)

seurat_normal7 <- CreateSeuratObject(counts = normal7_data,

project = "CON7",

min.cells = 3,

min.features = 200)

seurat_normal8 <- CreateSeuratObject(counts = normal7_data,

project = "CON8",

min.cells = 3,

min.features = 200)

seurat_normal9 <- CreateSeuratObject(counts = normal7_data,

project = "CON9",

min.cells = 3,

min.features = 200)

seurat_normal10 <- CreateSeuratObject(counts = normal7_data,

project = "CON10",

min.cells = 3,

min.features = 200)

seurat_normal11 <- CreateSeuratObject(counts = normal7_data,

project = "CON11",

min.cells = 3,

min.features = 200)

seurat_normal12 <- CreateSeuratObject(counts = normal7_data,

project = "CON12",

min.cells = 3,

min.features = 200)

seurat_normal13 <- CreateSeuratObject(counts = normal7_data,

project = "CON13",

min.cells = 3,

min.features = 200)

seurat_list <- list(seurat_cancer1, seurat_cancer2, seurat_cancer3, seurat_cancer4, seurat_cancer5, seurat_cancer6, seurat_cancer7, seurat_cancer8, seurat_cancer9, seurat_cancer10, seurat_cancer11, seurat_cancer12, seurat_cancer13, seurat_cancer14,seurat_normal1,seurat_normal2,seurat_normal3,seurat_normal4,seurat_normal5,seurat_normal6,seurat_normal7,seurat_normal8,seurat_normal9,seurat_normal10,seurat_normal11,seurat_normal12,seurat_normal13)

seurat_obj <- merge(seurat_list[[1]], y = seurat_list[-1])

table(seurat_obj$orig.ident)

seurat_obj[["percent.mt"]] <- PercentageFeatureSet(seurat_obj, pattern = "^MT-")

seurat_obj <- subset(seurat_obj,

subset = nFeature_RNA > 200 & nFeature_RNA < 7000 &

percent.mt < 20)

seurat_obj[["percent.rb"]] <- PercentageFeatureSet(seurat_obj, pattern = "^RP[SL]")

install.packages("ggplot2")

library(ggplot2)

qc_plot <- VlnPlot(seurat_obj,

features = c("nFeature_RNA", "nCount_RNA", "percent.mt", "percent.rb"),

ncol = 2, group.by = "orig.ident", pt.size = 0) +

ggtitle("figure 1")

seurat_obj <- subset(seurat_obj,

subset = nFeature_RNA > 200 &

nFeature_RNA < 6000 &

percent.mt < 20)

seurat_obj <- NormalizeData(seurat_obj)

seurat_obj <- FindVariableFeatures(seurat_obj, selection.method = "vst", nfeatures = 3000)

seurat_obj <- ScaleData(seurat_obj,

features = VariableFeatures(seurat_obj))

seurat_obj <- RunPCA(seurat_obj, features = VariableFeatures(seurat_obj))

pca_before <- DimPlot(seurat_obj, reduction = "pca", group.by = "orig.ident") +

ggtitle("figure 2A")

install.packages("devtools")

devtools::install_github("immunogenomics/harmony")

library(harmony)

seurat_obj <- RunHarmony(seurat_obj, group.by.vars = "orig.ident")

harmony_plot <- DimPlot(seurat_obj, reduction = "harmony", group.by = "orig.ident") +

ggtitle("figure 2B")

seurat_obj <- RunUMAP(seurat_obj, reduction = "harmony", dims = 1:30)

seurat_obj <- FindNeighbors(seurat_obj, reduction = "harmony", dims = 1:30)

seurat_obj <- FindClusters(seurat_obj, resolution = 0.8)

umap_clusters <- DimPlot(seurat_obj, reduction = "umap", label = TRUE, label.size = 4, pt.size = 0.5) +

ggtitle("UMAP - Seurat Clusters") +

theme(plot.title = element_text(hjust = 0.5, face = "bold", size = 14))

print(umap_clusters)

ggsave("UMAP_Clusters_Basic.png", umap_clusters, width = 10, height = 8)

cancer_markers <- c(

"CEACAM5", "CEACAM6", "MUC1", "CD44", "LGR5", "EPCAM",

"KRT8", "KRT18", "KRT19", "CDH1"

)

seurat_obj <- AddModuleScore(seurat_obj,

features = list(cancer_markers),

name = "cancer_score")

cancer_threshold <- median(seurat_obj$cancer_score1)

seurat_obj$tissue_type <- ifelse(seurat_obj$cancer_score1 > cancer_threshold, "cancer", "normal")

cat("RESULT:\n")

print(table(seurat_obj$tissue_type, seurat_obj$orig.ident))

cancer_classification_plot <- FeaturePlot(seurat_obj, features = "cancer_score1",

split.by = "orig.ident", order = TRUE) +

scale_color_viridis_c() +

ggtitle("figure 3")

install.packages("ggpubr")

library(ggpubr)

validation_genes <- c("MUC1", "CD44", "EPCAM",

"KRT8", "KRT18", "KRT19", "CDH1")

validation_plot <- VlnPlot(seurat_obj, features = validation_genes,

group.by = "tissue_type", ncol = 3, pt.size = 0) +

stat_compare_means(label = "p.signif") +

ggtitle("figure 4")

crc_markers <- list(

"Epithelial" = c("EPCAM", "CDH1", "KRT8", "KRT18", "KRT19","KRT20", "VIL1", "CDX2", "FABP1", "FABP2"),

"Cancer_cells" = c("CEACAM5", "CEACAM6", "MUC1", "CD44", "LGR5","CD133", "ALDH1A1", "MYC", "EGFR", "HER2","CTNNB1", "APC", "KRAS", "TP53", "PIK3CA"),

"Proliferation" = c("MKI67", "PCNA", "TOP2A", "CCNB1", "CCNB2",

"CCND1", "CCNE1", "CDK1", "CDK2", "CDK4"),

"T_cells" = c("CD3D", "CD3E", "CD4", "CD8A", "CD8B", "FOXP3",

"CD45", "CD2", "CD28", "PDCD1", "CTLA4", "LAG3",

"GZMB", "PRF1", "IFNG", "TNF", "IL2", "IL10"),

"B_cells" = c("CD19", "CD79A", "MS4A1", "CD27", "CD38",

"CD20", "CD22", "BLK", "PAX5", "CD24"),

"Macrophages" = c("CD68", "CD163", "MRC1", "ITGAM", "CSF1R",

"CD14", "CD86", "CD80", "CD206", "TREM2",

"CD64", "CD32", "IL1B", "TNF", "IL6"),

"Dendritic_cells" = c("CD1C", "CLEC9A", "XCR1", "CD141", "CD209",

"ITGAX", "CD11c", "HLA-DR", "CD83", "CD40"),

"Neutrophils" = c("FCGR3B", "CSF3R", "S100A8", "S100A9", "CXCR2",

"MPO", "ELANE", "CD177", "CD15", "CD66b"),

"Fibroblasts" = c("COL1A1", "COL3A1", "PDGFRA", "ACTA2", "FAP",

"COL1A2", "FN1", "TNC", "POSTN", "MMP2",

"MMP9", "TGFB1", "IL6", "CXCL12"),

"Endothelial" = c("PECAM1", "VWF", "CDH5", "CLDN5", "CD31",

"CD34", "KDR", "FLT1", "TEK", "SELE"),

"Mast_cells" = c("KIT", "TPSAB1", "CPA3", "MS4A2", "FCER1A",

"HDC", "CMA1", "CTSG"),

"Plasma_cells" = c("MZB1", "JCHAIN", "IGHA1", "IGHA2", "CD138",

"SDC1", "XBP1", "PRDM1", "IRF4"),

"Stem_cells" = c("LGR5", "ASCL2", "BMI1", "MSI1", "SOX9",

"OLFM4", "EPHB2", "TERT"),

"EMT" = c("VIM", "SNAI1", "SNAI2", "TWIST1", "ZEB1",

"ZEB2", "CDH2", "FN1", "MMP2", "MMP9"),

"Goblet_cells" = c("MUC2", "TFF3", "SPDEF", "KLF4", "FCGBP"),

"Paneth_cells" = c("LYZ", "DEF5", "DEFA6", "ANG4", "PLA2G2A")

)

library(ggplot2)

marker_dotplot <- DotPlot(seurat_obj, features = unique(unlist(crc_markers)),

group.by = "seurat_clusters") +

theme(axis.text.x = element_text(angle = 45, hjust = 1)) +

ggtitle("figure 5")

FeaturePlot(seurat_obj, features = unlist(crc_markers), ncol = 4)

new_cluster_ids <- c(

"0" = "T_cells", "1" = "Epithelial", "2" = "Fibroblasts",

"3" = "Macrophages", "4" = "Cancer_cells", "5" = "B_cells",

"6" = "Endothelial", "7" = "Dendritic_cells", "8" = "Mast_cells",

"9" = "Neutrophils", "10" = "Plasma_cells","11"= "Stem_cells",

"12" = "EMT","13" = "Goblet_cells","14" = "Paneth_cells"

)

current_clusters <- as.character(unique(seurat_obj$seurat_clusters))

undefined_clusters <- setdiff(current_clusters, names(new_cluster_ids))

clusters_char <- as.character(seurat_obj$seurat_clusters)

seurat_obj$celltype <- plyr::mapvalues(seurat_obj$seurat_clusters,

from = names(new_cluster_ids),

to = new_cluster_ids)

celltype_colors <- c(

"T_cells" = "#1F77B4",

"Epithelial" = "#FF7F0E",

"Fibroblasts" = "#2CA02C",

"Macrophages" = "#D62728",

"Cancer_cells" = "#9467BD",

"B_cells" = "#8C564B",

"Endothelial" = "#E377C2",

"Dendritic_cells" = "#7F7F7F",

"Mast_cells" = "#BCBD22",

"Neutrophils" = "#17BECF",

"Plasma_cells" = "#1F77B4",

"Stem_cells" = "#FF9896",

"EMT" = "#C5B0D5",

"Goblet_cells" = "#FFBB78",

"Paneth_cells" = "#98DF8A",

"^Unknown_" = "#D9D9D9"

)

celltype_umap <- DimPlot(seurat_obj, reduction = "umap",

group.by = "celltype",

label = FALSE,

label.size = 4,

repel = FALSE,

pt.size = 0.5,

raster = FALSE) +

scale_color_manual(values = celltype_colors) +

ggtitle("figure 6") +

theme_classic(base_size = 14) +

theme(

plot.title = element_text(hjust = 0.5, size = 16, face = "bold"),

axis.title = element_text(size = 14, face = "bold"),

axis.text = element_text(size = 12),

legend.title = element_text(size = 12, face = "bold"),

legend.text = element_text(size = 10),

legend.position = "right",

legend.key.size = unit(0.8, "cm"),

legend.box.spacing = unit(0.5, "cm"),

panel.border = element_rect(color = "black", fill = NA, size = 1)

) +

guides(color = guide_legend(override.aes = list(size = 3)))

#celltype_umap <- DimPlot(seurat_obj, reduction = "umap",

#group.by = "celltype", label = TRUE) +

#ggtitle("figure 6")

tissue_colors <- c(

"Tumor" = "#E41A1C",

"Normal" = "#377EB8",

"Metastasis" = "#4DAF4A",

"Adenoma" = "#984EA3"

)

tissue_celltype_plot <- DimPlot(seurat_obj, reduction = "umap",

group.by = "celltype",

split.by = "tissue_type",

label = FALSE,

pt.size = 0.4,

ncol = 2,

combine = TRUE) +

scale_color_manual(values = celltype_colors) +

ggtitle("figure 7") +

theme_minimal(base_size = 14) +

theme(

plot.title = element_text(hjust = 0.5, size = 18, face = "bold", margin = margin(b = 15)),

axis.title = element_text(size = 14, face = "bold"),

axis.text = element_text(size = 12),

strip.text = element_text(size = 14, face = "bold", margin = margin(5, 0, 5, 0)),

strip.background = element_rect(fill = "lightgray", color = "black", size = 0.5),

legend.title = element_text(size = 13, face = "bold", hjust = 0),

legend.text = element_text(size = 11),

legend.position = "right",

legend.key.size = unit(1, "cm"),

legend.key.height = unit(0.8, "cm"),

legend.spacing.y = unit(0.3, "cm"),

panel.border = element_rect(color = "black", fill = NA, size = 0.7),

panel.grid.major = element_blank(),

panel.grid.minor = element_blank(),

plot.margin = margin(15, 15, 15, 15)

) +

guides(color = guide_legend(override.aes = list(size = 4, alpha = 1)))

#tissue_celltype_plot <- DimPlot(seurat_obj, reduction = "umap",

#group.by = "celltype", split.by = "tissue_type") +

#ggtitle("figure 7")

trp_genes <- c(

"IDO1", "IDO2", "TDO2", "KYNU", "KMO", "HAAO", "QPRT",

"TPH1", "TPH2", "DDC", "MAOA", "MAOB", "SLC6A4",

"CYP1A1", "CYP1A2", "CYP1B1", "AHR", "ARNT",

"HTR1A", "HTR2A", "HTR3A", "HTR4", "HTR7",

"ALDH1A1", "ALDH1A2", "GOT1", "GOT2"

)

available_trp_genes <- trp_genes[trp_genes %in% rownames(seurat_obj)]

library(AUCell)

tryCatch({

expr_matrix <- LayerData(seurat_obj, layer = "data")

cells_rankings <- AUCell_buildRankings(expr_matrix)

trp_AUC <- AUCell_calcAUC(available_trp_genes, cells_rankings)

seurat_obj$trp_AUCell <- as.numeric(getAUC(trp_AUC))

cat("AUCell")

}, error = function(e) {

cat("way 1 failure:", e$message, "\n")

# way2

tryCatch({

temp_assay <- CreateAssay5Object(counts = LayerData(seurat_obj, layer = "counts"))

temp_assay <- SetAssayData(temp_assay, layer = "data",

new.data = LayerData(seurat_obj, layer = "data"))

expr_matrix <- GetAssayData(temp_assay, layer = "data")

cells_rankings <- AUCell_buildRankings(expr_matrix)

trp_AUC <- AUCell_calcAUC(available_trp_genes, cells_rankings)

seurat_obj$trp_AUCell <- as.numeric(getAUC(trp_AUC))

cat("AUCell success\n")

}, error = function(e2) {

cat("way2 failure:", e2$message, "\n")

# way3

cat("way3\n")

seurat_obj <- AddModuleScore(seurat_obj,

features = list(available_trp_genes),

name = "trp_metabolism")

seurat_obj$trp_AUCell <- seurat_obj$trp_metabolism1

})

})

if (!requireNamespace("BiocManager", quietly=TRUE))

install.packages("BiocManager")

BiocManager::install("UCell")

library(UCell)

tryCatch({

seurat_obj$trp_UCell <- UCell::ScoreSignatures_UCell(

matrix = LayerData(seurat_obj, layer = "data"),

features = list(TrpMetabolism = available_trp_genes)

)[,1]

cat("UCell success\n")

}, error = function(e) {

cat("UCell failure:", e$message, "\n")

seurat_obj$trp_UCell <- seurat_obj$trp_AUCell

})

# AddModuleScore

seurat_obj <- AddModuleScore(seurat_obj, features = list(trp_genes),

name = "trp_AddModuleScore")

expr_matrix <- as.matrix(LayerData(seurat_obj, layer = "data"))

tryCatch({

# way1

cat("way1\n")

gene_sets <- list(TrpPathway = available_trp_genes)

if(packageVersion("GSVA") >= "1.4.0") {

param <- gsvaParam(expr_matrix, gene_sets, min.sz=1, max.sz=Inf)

trp_ssGSEA <- gsva(param)

} else {

trp_ssGSEA <- gsva(expr_matrix, gene_sets, method="ssgsea",

min.sz=1, max.sz=Inf, verbose=TRUE)

}

seurat_obj$trp_ssGSEA <- as.numeric(trp_ssGSEA[1, ])

cat("GSVA success\n")

}, error = function(e) {

cat("way 1 failure:", e$message, "\n")

tryCatch({

cat("way2\n")

simple_ssgsea <- function(expr_mat, gene_set) {

gene_set <- gene_set[gene_set %in% rownames(expr_mat)]

if(length(gene_set) == 0) return(rep(0, ncol(expr_mat)))

ranks <- apply(expr_mat, 2, rank)

es <- apply(ranks, 2, function(col_ranks) {

in_geneset <- col_ranks[gene_set]

out_geneset <- col_ranks[setdiff(rownames(expr_mat), gene_set)]

if(length(in_geneset) > 0) {

sum(in_geneset) / length(in_geneset) - sum(out_geneset) / length(out_geneset)

} else {

0

}

})

return(es)

}

trp_ssGSEA_simple <- simple_ssgsea(expr_matrix, available_trp_genes)

seurat_obj$trp_ssGSEA <- trp_ssGSEA_simple

cat("way2 \n")

}, error = function(e2) {

cat("way2 failure:", e2$message, "\n")

cat("way3\n")

seurat_obj <- AddModuleScore(seurat_obj,

features = list(available_trp_genes),

name = "trp_module")

seurat_obj$trp_ssGSEA <- seurat_obj$trp_module1

})

})

score_columns <- c("trp_ssGSEA", "trp_AUCell", "trp_UCell", "trp_AddModuleScore")

available_scores <- score_columns[score_columns %in% colnames(seurat_obj@meta.data)]

colnames(seurat_obj@meta.data)

score_cols <- c("trp_AUCell", "trp_UCell", "trp_AddModuleScore1")

scores_df <- seurat_obj@meta.data[,score_cols, drop = FALSE]

head(scores_df)

summary(scores_df)

scores_df <- na.omit(scores_df)

cor_matrix <- cor(scores_df, method = "spearman")

print(cor_matrix)

write.csv(cor_matrix, "correlation_matrix.csv")

pdf("correlation_heatmap.pdf", width = 6, height = 5)

corrplot(cor_matrix, method = "color", type = "upper",

tl.col = "black", tl.srt = 45,

addCoef.col = "black", number.cex = 0.8,

title = "Correlation among scoring methods",

mar = c(0,0,2,0))

dev.off()

if(length(available_scores) > 0) {

seurat_obj$trp_avg_score <- rowMeans(seurat_obj@meta.data[, available_scores, drop = FALSE], na.rm = TRUE)

cat("JUDGE", paste(available_scores, collapse = ", "), "\n")

} else {

seurat_obj$trp_avg_score <- 0

cat("CATION\n")

}

celltype_table <- table(seurat_obj$celltype)

print(celltype_table)

known_celltypes <- c(

"T_cells", "Epithelial", "Fibroblasts", "Macrophages",

"Cancer_cells", "B_cells", "Endothelial", "Dendritic_cells",

"Mast_cells", "Neutrophils", "Plasma_cells", "Stem_cells",

"EMT", "Goblet_cells", "Paneth_cells"

)

existing_known_types <- intersect(known_celltypes, names(celltype_table))

cat("KNOWN", paste(existing_known_types, collapse = ", "), "\n")

unknown_celltypes <- grep("^Unknown_", names(celltype_table), value = TRUE)

cat("UNKNOW", length(unknown_celltypes), "\n")

cat("UNKNOW", sum(celltype_table[unknown_celltypes]),

sprintf("(%.1f%%)\n", 100 * sum(celltype_table[unknown_celltypes]) / sum(celltype_table)))

seurat_known <- subset(seurat_obj, subset = celltype %in% existing_known_types)

sprintf("(%.1f%%)\n", 100 * (ncol(seurat_obj) - ncol(seurat_known)) / ncol(seurat_obj)))

if (!"trp_avg_score" %in% colnames(seurat_known@meta.data)) {

if ("trp_avg_score" %in% colnames(seurat_obj@meta.data)) {

cat("CATION\n")

seurat_known <- AddMetaData(seurat_known,

metadata = seurat_obj$trp_avg_score[colnames(seurat_known)],

col.name = "trp_avg_score")

} else {

stop("WRONG")

}

}

library(viridis)

trp_activity_plot_clean <- FeaturePlot(seurat_known,

features = "trp_avg_score",

reduction = "umap",

split.by = "tissue_type",

label = FALSE,

repel = FALSE,

pt.size = 0.5,

order = TRUE,

combine = TRUE) +

scale_color_viridis_c(

option = "plasma",

na.value = "lightgray",

name = "TRP Activity\n(avg score)"

) +

ggtitle("figure 8") +

theme_minimal(base_size = 14) +

theme(

plot.title = element_text(hjust = 0.5, size = 16, face = "bold", margin = margin(b = 10)),

strip.text = element_text(size = 12, face = "bold"),

strip.background = element_rect(fill = "lightgray", color = "black", size = 0.5),

legend.title = element_text(size = 11, face = "bold"),

legend.text = element_text(size = 10),

legend.position = "right",

panel.border = element_rect(color = "black", fill = NA, size = 0.5),

panel.grid.major = element_blank(),

panel.grid.minor = element_blank(),

plot.margin = margin(10, 10, 10, 10)

)

trp_activity_plot_all <- FeaturePlot(seurat_obj,

features = "trp_avg_score",

reduction = "umap",

split.by = "tissue_type",

label = FALSE,

pt.size = 0.5,

order = TRUE) +

scale_color_viridis_c(

option = "plasma",

na.value = "lightgray",

name = "TRP Activity\n(avg score)"

) +

ggtitle("figure 8A) +

theme_minimal() +

theme(

plot.title = element_text(hjust = 0.5, size = 14, face = "bold"),

legend.position = "right"

)

library(patchwork)

comparison_plot <- trp_activity_plot_all / trp_activity_plot_clean +

plot_layout(heights = c(1, 1)) +

plot_annotation(

title = "title",

subtitle = "up | down ",

theme = theme(

plot.title = element_text(hjust = 0.5, size = 16, face = "bold"),

plot.subtitle = element_text(hjust = 0.5, size = 12)

)

)

trp_by_celltype <- aggregate(trp_avg_score ~ celltype,

data = seurat_known@meta.data,

FUN = mean)

trp_by_celltype <- trp_by_celltype[order(-trp_by_celltype$trp_avg_score), ]

colnames(trp_by_celltype) <- c("CellType", "Mean_TRP_Activity")

print(trp_by_celltype)

library(ggplot2)

trp_barplot <- ggplot(trp_by_celltype,

aes(x = reorder(CellType, Mean_TRP_Activity),

y = Mean_TRP_Activity,

fill = Mean_TRP_Activity)) +

geom_bar(stat = "identity", width = 0.7) +

scale_fill_viridis_c(

option = "plasma",

name = "TRP Activity"

) +

coord_flip() +

labs(

x = "cell symbol",

y = "average",

title = "figure 8B "

) +

theme_minimal(base_size = 12) +

theme(

plot.title = element_text(hjust = 0.5, size = 14, face = "bold"),

axis.text.y = element_text(size = 10),

axis.text.x = element_text(size = 10),

legend.position = "right",

panel.grid.major = element_blank(),

panel.grid.minor = element_blank(),

panel.border = element_rect(color = "black", fill = NA, size = 0.5)

) +

geom_text(

aes(label = sprintf("%.3f", Mean_TRP_Activity)),

hjust = -0.1,

size = 3.5,

fontface = "bold"

) +

scale_y_continuous(expand = expansion(mult = c(0, 0.1)))

trp_facet_by_celltype <- FeaturePlot(seurat_known,

features = "trp_avg_score",

reduction = "umap",

split.by = "celltype",

ncol = 4,

label = FALSE,

pt.size = 0.4,

order = TRUE) +

scale_color_viridis_c(

option = "plasma",

na.value = "lightgray",

name = "TRP Activity"

) +

ggtitle("figure 8C ") +

theme_minimal() +

theme(

plot.title = element_text(hjust = 0.5, size = 16, face = "bold"),

strip.text = element_text(size = 10, face = "bold"),

strip.background = element_rect(fill = "lightgray", color = "black"),

legend.position = "bottom",

legend.key.width = unit(1.5, "cm"),

panel.border = element_rect(color = "black", fill = NA, size = 0.5)

)

if (!exists("seurat_known")) {

known_celltypes <- c(

"T_cells", "Epithelial", "Fibroblasts", "Macrophages",

"Cancer_cells", "B_cells", "Endothelial", "Dendritic_cells",

"Mast_cells", "Neutrophils", "Plasma_cells", "Stem_cells",

"EMT", "Goblet_cells", "Paneth_cells"

)

existing_known_types <- intersect(known_celltypes, names(table(seurat_obj$celltype)))

seurat_known <- subset(seurat_obj, subset = celltype %in% existing_known_types)

sprintf("%.1f%%", 100 * ncol(seurat_known) / ncol(seurat_obj)), ")\n")

}

if (!"trp_avg_score" %in% colnames(seurat_known@meta.data)) {

if ("trp_avg_score" %in% colnames(seurat_obj@meta.data)) {

seurat_known <- AddMetaData(seurat_known,

metadata = seurat_obj$trp_avg_score[colnames(seurat_known)],

col.name = "trp_avg_score")

} else {

stop("wrong")

}

}

if (!requireNamespace("ggpubr", quietly = TRUE)) {

install.packages("ggpubr")

}

library(ggpubr)

celltype_trp_plot <- VlnPlot(seurat_known,

features = "trp_avg_score",

group.by = "celltype",

split.by = "tissue_type",

pt.size = 0,

adjust = 1,

log = FALSE,

fill.by = "split",

cols = c("#E41A1C", "#377EB8"),

sort = "decreasing") +

ggtitle("figure 9") +

theme_minimal(base_size = 12) +

theme(

plot.title = element_text(hjust = 0.5, size = 16, face = "bold", margin = margin(b = 15)),

axis.text.x = element_text(angle = 45, hjust = 1, size = 10, face = "bold"),

axis.text.y = element_text(size = 10),

axis.title = element_text(size = 12, face = "bold"),

legend.title = element_text(size = 11, face = "bold"),

legend.text = element_text(size = 10),

legend.position = "right",

panel.grid.major = element_line(color = "gray90", size = 0.2),

panel.grid.minor = element_blank(),

panel.border = element_rect(color = "black", fill = NA, size = 0.5),

plot.margin = margin(15, 15, 15, 15)

) +

labs(x = "cell sym", y = " avg score")

celltype_trp_plot_stats <- celltype_trp_plot +

stat_compare_means(

method = "wilcox.test",

label = "p.signif",

label.y = max(seurat_known$trp_avg_score, na.rm = TRUE) * 1.1,

size = 3.5,

hide.ns = TRUE,

vjust = 0.5

)

library(dplyr)

trp_summary <- seurat_obj@meta.data %>%

group_by(celltype, tissue_type) %>%

summarise(

mean_score = mean(trp_avg_score, na.rm = TRUE),

sd_score = sd(trp_avg_score, na.rm = TRUE),

n_cells = n()

)

write.csv(trp_summary, "results/Table1_trp_activity_summary.csv")

Idents(seurat_obj) <- "tissue_type"

seurat_obj <- JoinLayers(seurat_obj)

de_genes <- FindMarkers(seurat_obj,

ident.1 = "cancer", ident.2 = "normal",

logfc.threshold = 0.25,

min.pct = 0.1)

de_genes_significant <- de_genes[de_genes$p_val_adj < 0.05 & abs(de_genes$avg_log2FC) > 0.5, ]

write.csv(de_genes_significant, "results/Table2_differential_expression.csv")

top_de_genes <- rownames(de_genes_significant)[1:min(6, nrow(de_genes_significant))]

if(length(top_de_genes) > 0) {

de_heatmap <- DoHeatmap(seurat_obj, features = top_de_genes,

group.by = "tissue_type") +

ggtitle("figure 10 ")

}

library(clusterProfiler)

library(org.Hs.eg.db)

if(nrow(de_genes_significant) > 0) {

go_enrichment <- enrichGO(

gene = rownames(de_genes_significant),

OrgDb = org.Hs.eg.db,

keyType = "SYMBOL",

ont = "BP",

pAdjustMethod = "BH"

)

if(!is.null(go_enrichment) && nrow(go_enrichment) > 0) {

go_plot <- dotplot(go_enrichment, showCategory=15) +

ggtitle("figure 11 ")

write.csv(go_enrichment, "name.csv")

}

}

train_data <- read.table("GSE21815_expression_matrix.txt",sep = "\t",row.names = 1,check.names = F,stringsAsFactors = F,header = T)

train_clinical <- read.table("GSE21815_clinical_data.txt",

header = TRUE,

sep = "\t",

fill = TRUE,

stringsAsFactors = FALSE)

validation_data <- read.table("GSE103512_expression_matrix.txt",sep = "\t",row.names = 1,check.names = F,stringsAsFactors = F,header = T)

validation_clinical <- read.table("GSE103512_clinical_data.txt",

header = TRUE,

sep = "\t",

fill = TRUE,

stringsAsFactors = FALSE)

gse1 = getGEO('GSE21815', destdir=".", AnnotGPL = F, getGPL = F)

gse1[[1]]

clinical_data1 <- pData(gse1[[1]])

table(clinical_data1$title)

library(stringr)

group_list1 <- ifelse(

str_detect(clinical_data1$title,

regex("tumor|tumour|cancer|carcinoma|malignant|CRC", ignore_case = TRUE)),

"Tumor",

ifelse(

str_detect(clinical_data1$title,

regex("normal|control|healthy|non.tumor|non.tumour|benign", ignore_case = TRUE)),

"Normal",

"Unknown"

)

)

table(group_list1)

group_list1 <- factor(group_list1, levels = c("Tumor","Normal"))

expression_matrix1 <- read.table("expression_matrix1.txt",sep = "\t",row.names = 1,check.names = F,stringsAsFactors = F,header = T)

library(limma)

design=model.matrix(~group_list1)

fit=lmFit(expression_matrix1,design)

fit=eBayes(fit)

deg=topTable(fit,coef=2,number = Inf)

write.table(deg, file = "deg_all.txt",sep = "\t",row.names = T,col.names = NA,quote = F)

odd_cols <- seq(1, ncol(train_data), by = 2)

if (length(odd_cols) == 0) {

stop(" ")

}

train_data <- train_data[, odd_cols, drop = FALSE]

features <- t(train_data[trp_genes, ])

labels <- ifelse(train_clinical$source_name_ch1 == "colorectal cancer", 1, 0)

set.seed(123)

library('glmnet')

cv_lasso <- cv.glmnet(features, labels, family = "binomial", alpha = 1)

lasso_genes <- coef(cv_lasso, s = "lambda.min")

lasso_selected <- names(lasso_genes[lasso_genes[,1] != 0,])[-1]

install.packages("Boruta")

library('Boruta')

boruta_result <- Boruta(features, as.factor(labels), doTrace = 2)

boruta_selected <- getSelectedAttributes(boruta_result, withTentative = TRUE)

install.packages("caret")

library('caret')

class(features)

class(labels)

ctrl <- rfeControl(functions = caretFuncs, method = "cv", number = 5)

svm_profile <- rfe(features, as.factor(labels),

sizes = c(1:10),

rfeControl = ctrl,

method = "svmRadial")

svm_selected <- predictors(svm_profile)

# GBM (Gradient Boosting Machine)

install.packages("gbm")

library('gbm')

gbm_model <- gbm(labels ~ ., data = data.frame(features),

distribution = "bernoulli",

n.trees = 1000,

interaction.depth = 3,

shrinkage = 0.01,

cv.folds = 5)

gbm_importance <- summary(gbm_model, plotit = FALSE)

gbm_selected <- gbm_importance$var[1:10]

install.packages("randomForest")

library('randomForest')

rf_model <- randomForest(features, as.factor(labels), importance = TRUE)

rf_importance <- importance(rf_model)

rf_selected <- rownames(rf_importance)[order(rf_importance[, "MeanDecreaseGini"],

decreasing = TRUE)[1:10]]

all_selected <- list(

LASSO = lasso_selected,

Boruta = boruta_selected,

#SVM_RFE = svm_selected,

GBM = gbm_selected,

RandomForest = rf_selected

)

library(VennDiagram)

venn_plot <- venn.diagram(all_selected, filename = NULL, fill = rainbow(4))

grid.draw(venn_plot)

common_genes <- Reduce(intersect, all_selected)

print(paste("name", paste(common_genes, collapse = ", ")))

library(ggplot2)

library(glmnet)

library(Boruta)

library(caret)

library(gbm)

library(randomForest)

library(pROC)

library(pheatmap)

library(RColorBrewer)

library(ggpubr)

library(reshape2)

my_colors <- brewer.pal(4, "Set2")

names(my_colors) <- c("LASSO", "Boruta", "GBM", "RandomForest")

png("name.png", width = 1000, height = 800)

par(mfrow = c(1, 2), mar = c(5, 5, 4, 2))

plot(cv_lasso$glmnet.fit, xvar = "lambda",

main = "name",

xlab = "Log Lambda",

ylab = "Coefficients",

col = my_colors[1],

lwd = 2)

plot(cv_lasso,

main = "LASSO",

xlab = "Log Lambda",

ylab = "Binomial Deviance",

col = my_colors[1],

lwd = 2)

abline(v = log(cv_lasso$lambda.min), lty = 2, col = "red", lwd = 2)

abline(v = log(cv_lasso$lambda.1se), lty = 2, col = "blue", lwd = 2)

legend("topright",

legend = c("λ.min", "λ.1se"),

col = c("red", "blue"),

lty = 2, lwd = 2)

dev.off()

if(length(lasso_selected) > 0) {

tryCatch({

coef_values <- as.numeric(lasso_genes[lasso_genes[,1] != 0 & rownames(lasso_genes) != "(Intercept)", 1])

gene_names <- rownames(lasso_genes)[lasso_genes[,1] != 0 & rownames(lasso_genes) != "(Intercept)"]

lasso_importance <- data.frame(

Gene = gene_names,

Coefficient = coef_values

)

lasso_importance <- lasso_importance[order(abs(lasso_importance$Coefficient), decreasing = TRUE), ]

p_lasso_bar <- ggplot(lasso_importance, aes(x = reorder(Gene, Coefficient), y = Coefficient)) +

geom_bar(stat = "identity", fill = my_colors[1], alpha = 0.8) +

coord_flip() +

theme_minimal(base_size = 14) +

theme(

plot.title = element_text(hjust = 0.5, face = "bold", size = 16),

axis.text.y = element_text(face = "bold")

) +

labs(

title = "LASSO ",

x = "gene",

y = "LASSO "

)

ggsave("LASSO_Selected_Genes.png", p_lasso_bar, width = 10, height = max(6, nrow(lasso_importance)/2))

print(p_lasso_bar)

cat("LASSO SAVED \n")

}, error = function(e) {

cat("LASSO failure:", e$message, "\n")

})

} else {

cat("LASSOnone\n")

}

png("Boruta_Importance.png", width = 1000, height = 800)

par(mar = c(10, 5, 4, 2))

boruta_importance <- attStats(boruta_result)

boruta_importance <- boruta_importance[order(boruta_importance$meanImp, decreasing = TRUE), ]

colors <- ifelse(boruta_importance$decision == "Confirmed", "green",

ifelse(boruta_importance$decision == "Tentative", "yellow", "red"))

barplot(boruta_importance$meanImp[1:min(20, nrow(boruta_importance))],

names.arg = rownames(boruta_importance)[1:min(20, nrow(boruta_importance))],

col = colors[1:min(20, nrow(boruta_importance))],

las = 2,

cex.names = 0.8,

ylab = "ave Z-score",

main = "Boruta ")

legend("topright",

legend = c("Confirmed", "Tentative", "Rejected"),

fill = c("green", "yellow", "red"),

cex = 0.8)

dev.off()

png("Boruta_Decision_Boundary.png", width = 1200, height = 600)

par(mfrow = c(1, 2), mar = c(5, 5, 4, 2))

plot(boruta_result,

cex.axis = 0.7,

las = 2,

xlab = "",

main = "Boruta")

boxplot(boruta_result,

las = 2,

cex.axis = 0.7,

main = "Boruta ")

dev.off()

create_gbm_plots <- function() {

tryCatch({

if(exists("gbm_importance") && nrow(gbm_importance) > 0) {

p_gbm_importance <- ggplot(gbm_importance, aes(x = reorder(var, rel.inf), y = rel.inf)) +

geom_bar(stat = "identity", fill = my_colors[4], alpha = 0.8) +

coord_flip() +

theme_minimal(base_size = 14) +

theme(

plot.title = element_text(hjust = 0.5, face = "bold", size = 16),

axis.text.y = element_text(face = "bold")

) +

labs(

title = "GBM ",

x = "gene",

y = " "

)

ggsave("GBM_Feature_Importance.png", p_gbm_importance,

width = 10, height = max(6, nrow(gbm_importance)/3))

print(p_gbm_importance)

}

}, error = function(e) {

})

}

create_gbm_plots()

create_gbm_learning_curve <- function() {

tryCatch({

png("GBM_Learning_Curve.png", width = 800, height = 600)

par(mar = c(5, 5, 4, 2))

best_iter <- gbm.perf(gbm_model, method = "cv", plot.it = FALSE)

plot(gbm_model$cv.error,

type = "l",

col = my_colors[4],

lwd = 2,

xlab = " ",

ylab = " ",

main = " ")

if(!is.na(best_iter)) {

abline(v = best_iter, lty = 2, col = "red", lwd = 2)

legend("topright",

legend = paste("best time:", best_iter),

col = "red",

lty = 2,

lwd = 2)

}

dev.off()

}, error = function(e) {

})

}

create_gbm_learning_curve()

create_rf_plots <- function() {

tryCatch({

rf_importance_df <- as.data.frame(rf_importance)

rf_importance_df$Gene <- rownames(rf_importance_df)

rf_importance_df <- rf_importance_df[order(rf_importance_df$MeanDecreaseGini, decreasing = TRUE), ]

rf_importance_top <- head(rf_importance_df, 15)

p_rf_importance <- ggplot(rf_importance_top, aes(x = reorder(Gene, MeanDecreaseGini), y = MeanDecreaseGini)) +

geom_bar(stat = "identity", fill = my_colors[5], alpha = 0.8) +

coord_flip() +

theme_minimal(base_size = 14) +

theme(

plot.title = element_text(hjust = 0.5, face = "bold", size = 16),

axis.text.y = element_text(face = "bold")

) +

labs(

title = "Top Genes",

x = "gene",

y = "average"

)

ggsave("RandomForest_Feature_Importance.png", p_rf_importance, width = 10, height = 8)

print(p_rf_importance)

}, error = function(e) {

})

}

create_rf_plots()

create_rf_error_plot <- function() {

tryCatch({

png("RandomForest_Error_Rate.png", width = 1000, height = 600)

par(mar = c(5, 5, 4, 2))

if(!is.null(rf_model$err.rate) && nrow(rf_model$err.rate) > 0) {

matplot(1:nrow(rf_model$err.rate), rf_model$err.rate,

type = "l",

lty = 1,

col = c("black", "red", "green"),

xlab = "amount",

ylab = "error rate",

main = "change",

lwd = 2)

legend("topright",

legend = c("OOB error rate", "Control error rate", "Tumor error rate"),

col = c("black", "red", "green"),

lty = 1,

lwd = 2)

}

dev.off()

}, error = function(e) {

})

}

create_rf_error_plot()

train_predictions <- predict(rf_model, features)

install.packages("pROC")

library(pROC)

train_roc <- roc(labels, as.numeric(train_predictions))

plot(train_roc, main = "Training Set ROC Curve")

validation_data <- validation_data[, -(1:75)]

validation_data <- validation_data[, -(70:205)]

validation_features <- t(validation_data[common_genes, ])

validation_labels <- ifelse(validation_clinical$characteristics_ch1.1 == "normal: no", 1, 0)

feature_names <- colnames(features)

safe_feature_alignment <- function(validation_features, feature_names) {

if(!is.data.frame(validation_features)) {

validation_features <- as.data.frame(validation_features)

}

current_features <- colnames(validation_features)

missing_features <- setdiff(feature_names, current_features)

if(length(missing_features) > 0) {

}

extra_features <- setdiff(current_features, feature_names)

aligned_data <- data.frame(matrix(

nrow = nrow(validation_features),

ncol = length(feature_names)

))

colnames(aligned_data) <- feature_names

common_features <- intersect(feature_names, current_features)

if(length(common_features) > 0) {

aligned_data[, common_features] <- validation_features[, common_features, drop = FALSE]

}

if(length(missing_features) > 0) {

for(feature in missing_features) {

aligned_data[[feature]] <- 0

}

}

return(aligned_data)

}

validation_features_aligned <- safe_feature_alignment(validation_features, feature_names)

validation_predictions <- predict(rf_model, validation_features_aligned)

validation_roc <- roc(validation_labels, as.numeric(validation_predictions))

plot(validation_roc, main = "Validation Set ROC Curve", col = "blue")

ego <- enrichGO(gene = common_genes,

OrgDb = org.Hs.eg.db,

keyType = "SYMBOL",

ont = "BP",

pAdjustMethod = "BH",

qvalueCutoff = 0.05)

dotplot(ego, showCategory = 15)

install.packages("devtools")

options(download.file.method = "libcurl")

options(timeout = 400)

devtools::install_github("sqjin/CellChat")

install.packages("parallelly")

install.packages("systemfonts")

devtools::install_github("sqjin/CellChat")

library(CellChat)

cellchat_obj <- createCellChat(object = seurat_obj, group.by = "seurat_clusters")

cellchat_obj <- identifyOverExpressedGenes(cellchat_obj)

cellchat_obj <- identifyOverExpressedInteractions(cellchat_obj)

cellchat_obj <- computeCommunProb(cellchat_obj)

cellchat_obj <- aggregateNet(cellchat_obj)

data.input <- GetAssayData(seurat_obj, layer = "data", assay = "RNA")

identity <- data.frame(ident = Idents(seurat_obj), row.names = colnames(seurat_obj))

cellchat <- createCellChat(object = data.input, meta = identity, group.by = "ident")

cellchat <- addMeta(cellchat, meta = identity)

cellchat <- setIdent(cellchat, ident.use = "ident")

CellChatDB <- CellChatDB.human

cellchat@DB <- CellChatDB

cellchat <- subsetData(cellchat)

cellchat <- identifyOverExpressedGenes(cellchat)

cellchat <- identifyOverExpressedInteractions(cellchat)

cellchat <- computeCommunProb(cellchat)

optimal_genes <- readLines("optimal_genes.txt")

cellchat <- identifyOverExpressedGenes(cellchat, features = optimal_genes)

cellchat <- computeCommunProb(cellchat, trim = 0.1)

my_genes <- read.table("optimal_genes.txt")$V1

cellchat <- filterCommunication(cellchat, ligand = my_genes, receptor = my_genes)

cellchat <- computeCommunProbPathway(cellchat)

cellchat <- aggregateNet(cellchat)

netVisual_aggregate(cellchat, signaling = "all", layout = "circle")

netVisual_circle(cellchat_obj@net$count, vertex.weight = groupSize)

install.packages("devtools")

if (!require("remotes", quietly = TRUE)) install.packages("remotes")

install.packages("D:/Download/grr_0.9.2.tar.gz",

repos = NULL,

type = "source")

remotes::install_github("brentp/Matrix.utils")

remotes::install_github('cole-trapnell-lab/leidenbase')

remotes::install_github('cole-trapnell-lab/monocle3')

library(monocle3)

cds <- as.CellDataSet(seurat_obj)

cds <- estimateSizeFactors(cds)

cds <- estimateDispersions(cds)

cds <- reduceDimension(cds, max_components = 2, method = 'DDRTree')

cds <- orderCells(cds)

plot_cell_trajectory(cds, color_by = "Pseudotime")
